# Multi-omics integration in malignant pleural mesothelioma: from molecular evolution and immune ecosystems to precision therapy

**DOI:** 10.3389/fonc.2026.1883870

**Published:** 2026-08-13

**Authors:** Hongmei Li, Kunzhong Zhou, Ruiting Xu, Weifang Wang, Tingting Song, Yonghong Zhou, Jing Ren, Zhiyong Wang, Yiteng Liang, Yilin Chen, Saurav Poudel, Junxuan Yu, Rui Lin, Ke Yin, Shengjie Yang, Li Zhuang

**Affiliations:** 1 Department of Rehabilitation and Palliative Medicine, The Third Affiliated Hospital of Kunming Medical University, Yunnan Cancer Hospital, Peking University Cancer Hospital Yunnan, Kunming, China; 2 Puer Central Hospital, Simao District People’s Hospital, Puer, China; 3 Department of Thoracic Surgery, The People’s Hospital of Chu Xiong Yi Autonomous Prefecture, Chu Xiong, China

**Keywords:** immune ecosystem stratification, malignant pleural mesothelioma, multi-omics, precision therapy, single-cell sequencing, spatial omics, tumor microenvironment

## Abstract

Malignant pleural mesothelioma (MPM) exhibits substantial molecular and cell-state heterogeneity, together with marked spatial variation in its immune and stromal microenvironment. Histological classification and individual biomarkers alone cannot adequately explain its evolution or heterogeneous treatment responses. This review critically integrates genomic, epigenomic, transcriptomic, single-cell, and spatial omics evidence along a conceptual continuum encompassing tumor-intrinsic molecular evolution, microenvironmental ecosystem remodeling, candidate stratification, and precision therapy. Current evidence indicates that alterations involving *BAP1, CDKN2A/MTAP*, and the *NF2*/Hippo pathway impose evolutionary constraints and create candidate therapeutic vulnerabilities but are insufficient to determine functional phenotypes independently. Epithelioid, uncommitted, and sarcomatoid malignant cell states may coexist within the same genetic clone, while immune and stromal features display pronounced regional heterogeneity. Based on published human MPM cohorts, we further propose four provisional immune-ecosystem archetypes: T-cell–inflamed, B-cell/tertiary lymphoid structure–organized, myeloid-dominant, and immune-desert. Tumor-intrinsic molecular states and stromal–spatial features are treated as interpretive modifiers. This literature-derived framework is intended for hypothesis generation and has not been developed or validated as a clinical classifier. At present, the principal value of multi-omics lies in mechanistic discovery and the design of biomarker-enriched clinical trials. Future studies should employ standardized assays in multicenter cohorts, independent external validation, formal archetype-by-treatment interaction testing within randomized trials, and longitudinal sampling to determine the treatment-predictive value and clinical feasibility of this framework.

## 1 Introduction: from histological classification to a molecular–immune ecosystem perspective on MPM

### 1.1 Clinical burden and biological heterogeneity of MPM

Malignant pleural mesothelioma (MPM) is a rare and aggressive malignancy arising from pleural mesothelial cells. It is characterized by a prolonged latency period, rapid disease progression, and limited therapeutic responsiveness. In 2022, approximately 30,618 new cases of malignant mesothelioma were diagnosed worldwide, accounting for 0.2% of all newly diagnosed cancers (1). Although these estimates also include mesotheliomas arising from the peritoneum, pericardium, and other sites and therefore cannot be equated directly with the incidence of MPM, previous cohort studies suggest that more than 80% of mesotheliomas arise from the pleura (2). These estimates therefore provide an approximate indication of the global disease burden. Despite advances in therapeutic strategies, the overall prognosis of MPM remains poor. Median survival varies according to disease stage, histological subtype, and treatment modality, but historically has ranged from 8 to 14 months, with a 5-year survival rate below 10% (3, 4).

Asbestos exposure remains the principal established risk factor for MPM (3, 5). Nevertheless, marked geographic variation in disease incidence suggests that MPM is not driven by a single environmental factor (6, 7). Instead, its development reflects the combined effects of environmental exposure, genetic susceptibility, genomic alterations, epigenetic dysregulation, and remodeling of the tumor microenvironment (TME).

Histopathologically, MPM is classified into epithelioid (50%–60%), sarcomatoid (7%–10%), and biphasic subtypes (20%–30%) (8), although the reported proportions vary according to sampling method, patient selection, and pathological assessment. Patients with epithelioid MPM have historically shown a relatively favorable median survival of approximately 14 months, whereas those with the more aggressive sarcomatoid subtype have a median survival of approximately 4 months (4). Histological classification has therefore long served as a foundation for MPM diagnosis, prognostic assessment, and treatment decision-making. However, substantial variation in molecular states, disease progression, immune ecosystems, and therapeutic responses persists within individual histological subtypes, indicating that morphology alone cannot fully capture the biological heterogeneity of MPM.

### 1.2 Limitations of current classification and the role of multi-omics integration

The early clinical manifestations of MPM are nonspecific (9), and most patients are diagnosed with advanced-stage disease. Histopathological examination remains central to diagnosis; however, the diffuse growth pattern along the pleura, limited biopsy material, and pronounced intratumoral heterogeneity restrict the ability of a single-site specimen to represent the disease as a whole. Although established immunohistochemical markers have improved diagnostic accuracy (3, 8), they remain insufficient for reliable molecular stratification or prediction of therapeutic response (10).

Dual immune checkpoint blockade and chemoimmunotherapy have reshaped the systemic treatment landscape for unresectable MPM. In the phase III CheckMate 743 trial, first-line nivolumab plus ipilimumab significantly prolonged median overall survival compared with platinum–pemetrexed chemotherapy in previously untreated patients with unresectable MPM, thereby establishing dual immune checkpoint blockade as a first-line treatment option (11). Nevertheless, durable benefit remains confined to a subset of patients, and treatment responses vary substantially among individuals. The absence of robust and reproducible predictive biomarkers further indicates that histological classification and a limited number of individual molecular markers are insufficient to guide precision treatment.

Against this background, this review adopts the molecular–immune ecosystem as its organizing concept and critically integrates genomic, epigenomic, transcriptomic, single-cell, and spatial transcriptomic evidence across MPM initiation, microenvironmental remodeling, and precision therapy. We further synthesize immune states recurrently observed across independent human cohorts into four provisional archetypes—T-cell–inflamed, B-cell/tertiary lymphoid structure–organized, myeloid-dominant, and immune-desert—to provide a structured basis for generating testable hypotheses regarding patient stratification and treatment matching.

## 2 Multi-omics dissection of tumor-intrinsic molecular evolution in MPM

### 2.1 Environmental exposure and genetic susceptibility initiate malignant transformation

The development of MPM is not driven by a single etiologic factor but reflects the combined effects of environmental exposure, genetic susceptibility, and multilayered molecular abnormalities. Asbestos exposure remains the environmental risk factor supported by the most robust evidence (3, 5, 7), whereas erionite exposure (12, 13) and ionizing radiation (14) contribute substantially less to the overall disease burden. Experimental studies have shown that asbestos fibers can persist in the pleura for prolonged periods and promote chronic inflammation through activation of the NLRP3 inflammasome and NF-κB signaling (15–19). Asbestos exposure can also induce macrophage-derived reactive oxygen species, leading to DNA damage and genomic instability (20, 21).

However, not all asbestos-exposed individuals develop MPM, indicating that host genetic background can modify individual susceptibility to environmental exposure. Germline pathogenic variants in *BAP1* are among the best-supported inherited susceptibility factors, with one study estimating that carriers have a higher risk of developing mesothelioma than the general population (22). In germline Bap1-mutant mice, low-dose exposure to amphibole or serpentine asbestos increased mesothelioma development and was accompanied by an immunosuppressive tumor microenvironment (23). These findings support a gene–environment interaction in a specific genetically susceptible background. Nevertheless, because the evidence derives from a preclinical model, it should not be generalized to all patients with MPM.

Simian virus 40 (SV40) has also been proposed as a potential co-carcinogenic factor. Early tissue-based and serologic studies reported positive findings in subsets of patients, and some studies suggested that SV40 might modify asbestos-associated risk (24–29). However, its relationship with MPM remains controversial, with marked geographic heterogeneity across studies. Investigations conducted in the asbestos-polluted region of Dayao County in Yunnan, China, and in Japan found no clear association between SV40 and MPM (30, 31). Most available studies have relied on viral-marker detection or serologic associations and are limited by differences in sample origin, analytical methods, and regional exposure patterns. Large, multicenter prospective studies are lacking. Consequently, a cooperative role for SV40 under specific environmental or genetic conditions cannot be completely excluded, but current evidence does not support SV40 as a common independent cause of MPM. By contrast, asbestos-induced chronic inflammation, oxidative stress, and genomic injury are supported by more consistent epidemiologic and experimental evidence and remain the principal established environmental mechanisms underlying MPM. These sustained selective pressures provide the initial context for subsequent molecular evolution.

### 2.2 Genomic and epigenomic vulnerabilities

Genomic and epigenomic studies have shown that MPM lacks highly recurrent, directly targetable oncogenic mutations analogous to those observed in several other solid tumors. Its molecular landscape is instead dominated by the inactivation of tumor suppressor genes, including *BAP1, CDKN2A/CDKN2B, NF2, TP53*, and *LATS2* (32–34), together with abnormalities in chromatin regulation and cell-cycle control. Tumor-intrinsic heterogeneity in MPM is determined not only by which genes are altered but also by the timing, clonality, and spatial distribution of these events and by the functional consequences of structural variation and epigenetic states. Cataloguing mutation frequencies alone is therefore insufficient to explain the evolutionary trajectories or therapeutic vulnerabilities of MPM.

#### 2.2.1 *BAP1* inactivation and chromatin dysregulation

*BAP1* is among the most frequently inactivated genes in MPM, although its reported prevalence varies considerably according to cohort composition, analytical platform, and the definition of inactivation. In addition to germline pathogenic variants, sporadic MPM may harbor somatic mutations, copy-number deletions, or structural variants that disrupt *BAP1 (*32, 33). The gene encodes a deubiquitinase involved in chromatin regulation and the DNA-damage response, and its loss can promote both genomic and epigenomic instability (32, 35, 36).

Multisite sampling studies have shown that *BAP1* alterations are clonally distributed in most evaluated tumors, supporting their potential role as relatively early evolutionary events (37, 38). However, this pattern is not restricted to a specific histological subtype. Whole-genome sequencing (WGS) has further demonstrated that structural variants and copy-number alterations can identify *BAP1* inactivation events that may be missed by whole-exome sequencing (WES). Deletions involving chromosome 3p21 may also encompass the neighboring gene *PBRM1*. Such co-deletion primarily reflects chromosomal proximity, and whether the simultaneous loss of these genes produces functional cooperation remains uncertain (32).

A large next-generation sequencing analysis of pleural and peritoneal mesotheliomas stratified tumors into four groups according to *BAP1* and *CDKN2A/CDKN2B* alteration status: *BAP1* alteration alone, *CDKN2A/CDKN2B* alteration alone, concurrent alterations, and neither alteration detected. These groups exhibited distinct co-mutation and copy-number profiles (33). The findings suggest that mesotheliomas may evolve along different molecular trajectories rather than through a uniform oncogenic process. However, this classification remains investigational, and its prognostic and treatment-predictive value requires validation in independent cohorts.

#### 2.2.2 *CDKN2A/MTAP* loss and cell-cycle–metabolic vulnerability

Homozygous deletion of *CDKN2A* at chromosome 9p21 disrupts both the p16INK4A–CDK4/6–RB and p14ARF–MDM2–p53 regulatory axes (32, 33, 39). Its reported prevalence varies according to the detection method and histological composition of the study population and should not be extrapolated from individual cohorts to all patients with MPM.

Because of their genomic proximity, *CDKN2A* and *MTAP* are frequently co-deleted. *MTAP* is involved in the clearance of 5′-methylthioadenosine (MTA); its loss results in abnormal MTA accumulation and perturbs methyl-donor metabolism, thereby creating a selective dependence on PRMT5-mediated methylation (40). This mechanism extends the biological consequences of 9p21 deletion beyond cell-cycle dysregulation to include a metabolic–epigenetic vulnerability and provides a rationale for therapeutic strategies targeting PRMT5-related pathways (41–43). However, the available evidence is derived predominantly from preclinical models and early-phase clinical studies. Whether *MTAP* loss can serve as a reproducible predictive biomarker and whether targeting this dependency improves clinical outcomes in MPM remain to be established prospectively.

#### 2.2.3 *NF2*/hippo pathway dysregulation and clonal heterogeneity

*NF2* encodes Merlin, an upstream regulator of the Hippo signaling pathway. Its inactivation can promote aberrant YAP/TAZ activation and thereby enhance cell proliferation, survival, and invasion (32, 44). Less frequent alterations in other Hippo pathway components, including *LATS2*, may produce similar effects, indicating that pathway dysregulation is not restricted to *NF2* loss (44, 45).

Among the canonical driver alterations in MPM, *NF2* inactivation was historically regarded as an early clonal event. However, a study based on paired biopsies from multiple anatomic sites challenged this interpretation. Among 16 patients subjected to multiregional analysis, eight harbored *NF2* mutations, three of whom showed heterogeneous distribution of these mutations across different anatomic sites. By contrast, alterations in genes such as *BAP1* and *SETD2* were comparatively stable (37). These findings suggest that, in a subset of patients, *NF2* inactivation may be acquired by specific subclones after tumor establishment rather than universally representing an initiating event. Nevertheless, the evidence was derived from a small exploratory cohort, and the prevalence and biological significance of subclonal *NF2* alterations require confirmation in independent studies.

#### 2.2.4 Structural variation and genome-wide instability

WGS has expanded the genomic landscape of MPM beyond point mutations to encompass structural variants, copy-number alterations, and abnormalities in ploidy. Earlier integrative exome–transcriptome studies demonstrated that gene fusions and aberrant splicing represent important mechanisms of tumor-suppressor inactivation, affecting genes such as *NF2*, *BAP1*, and *SETD2*. The Cancer Genome Atlas (TCGA) integrative analysis likewise revealed extensive copy-number losses and loss of heterozygosity in MPM (46, 47). Subsequently, a WGS study of 58 MPM tumors, combined with an integrated analysis of data from 229 patients, showed that MPM is characterized by a generally low tumor mutational burden but widespread structural variation, which can further disrupt key tumor-suppressor genes, including *BAP1*, *NF2*, and *TP53* (32). The MESOMICS study further showed that MPM harbors a low burden of small variants but a relatively high structural-variant burden compared with other tumor types with similar mutational burdens. Approximately 20% of samples exhibited complex rearrangement patterns consistent with chromosomal shattering, and some of these rearrangements generated fusion transcripts detectable at the transcriptional level (48). Collectively, these cross-platform findings indicate that genomic damage in MPM is manifested predominantly through copy-number and structural alterations rather than through the accumulation of point mutations alone. Accordingly, analyses based solely on whole-exome sequencing or targeted sequencing may underestimate the true extent of genomic disruption. However, because the available studies differ in sample type, sequencing depth, and structural-variant detection algorithms, their reported event frequencies should not be compared directly.

Ploidy alterations may represent an additional and partially independent dimension of genomic heterogeneity. Among the 58 WGS samples, whole-genome doubling (WGD) was detected in 17 tumors (29%) and was associated with shorter overall survival in an unadjusted analysis (log-rank *p* < 0.01) (32). The MESOMICS study further identified a continuous ploidy axis extending from genome-wide near-haploidization to WGD and observed an association between WGD status and prognosis in an external cohort (48). These convergent findings support the potential prognostic relevance of ploidy status. Nevertheless, current evidence is insufficient to establish whether WGD provides prognostic information independent of histological subtype, disease stage, and other clinical variables. WGD should therefore be considered a candidate prognostic feature rather than an established independent biomarker.

*RBFOX1* was recurrently affected by structural-variant breakpoints in the aforementioned WGS cohort of 58 tumors and was also among the genes frequently involved in structural alterations in the MESOMICS cohort (32, 48). This cross-cohort recurrence supports *RBFOX1* as a candidate genomic region that is repeatedly disrupted in MPM, but it does not establish a selectively advantageous driver role. Similarly, *PBRM1* may be co-deleted with *BAP1*; however, because the two genes are located in close proximity within the 3p21 region, their co-deletion may primarily reflect a shared chromosomal event. Whether *PBRM1* loss provides biological or therapeutic information independent of *BAP1* loss remains to be determined through functional studies.

Evidence regarding homologous recombination deficiency (HRD) remains inconsistent. When stringent dual criteria based on both HRDetect and HRD scores were applied, only 2 of the 58 WGS tumors exhibited both high HRD scores and evidence consistent with functional impairment of BRCA1/2 (32). By contrast, the MESOMICS study classified approximately 23% of samples as exhibiting an HRD phenotype on the basis of copy-number features or structural-variant patterns (48). This discrepancy may reflect differences in analytical algorithms, classification thresholds, and operational definitions of HRD. It also underscores that a positive genomic-scar signature does not necessarily indicate an ongoing functional deficiency in BRCA1/2 or sensitivity to poly(ADP-ribose) polymerase (PARP) inhibitors. Current evidence therefore does not support the unselected use of PARP inhibitors in patients with MPM. Nevertheless, it provides a rationale for prospective evaluation in molecularly defined subgroups in which HRD has been confirmed using standardized approaches. The effects of relatively infrequent alterations in *TP53*, *LATS2*, and *SETD2* should likewise be interpreted in the context of co-occurring alterations, structural variation, and clonal architecture, rather than inferring biological behavior or therapeutic response from single-gene status alone.

#### 2.2.5 Epigenetic remodeling and plastic molecular states

Beyond genomic mutations, aberrant DNA methylation, altered histone modifications (49–52), and dysregulated noncoding RNAs (53–55) can reshape transcriptional programs involved in tumor-cell proliferation, invasion, and immune evasion. The MESOMICS study integrated genomic, transcriptomic, and DNA-methylation data from 120 patients with MPM and identified a CpG island methylator phenotype (CIMP) axis that was independent of histological classification. The CIMP-high state was characterized by increased *EZH2* expression, promoter hypermethylation and transcriptional repression of polycomb repressive complex 2 (PRC2) target genes, including *WT1*, and an association with poorer prognosis (48). These findings demonstrate that histologically similar tumors may occupy distinct epigenetic states. However, CIMP remains an investigational stratification variable rather than an established clinical classification criterion.

In cell-line models, the CIMP-low state was associated with greater sensitivity to olaparib, whereas *BAP1* status alone did not consistently predict response to poly(ADP-ribose) polymerase inhibition (48). This observation suggests that a functional epigenetic state may more closely reflect a drug-response phenotype than an individual genomic alteration. Nevertheless, the threshold used to define CIMP status and its treatment-predictive value require validation in prospective clinical studies.

At the level of histone modification, aberrant expression or activity of chromatin-modifying enzymes can substantially alter chromatin organization and gene transcription. KDM4A overexpression, SETDB1 inactivation, and increased EZH2 activity can disrupt H3K9- or H3K27-associated methylation and thereby affect gene expression and chromatin stability (48, 56, 57). The EZH2 inhibitor tazemetostat has demonstrated modest antitumor activity in patients with *BAP1*-inactivated MPM (58), supporting the biological feasibility of epigenetic targeting. However, these findings are insufficient to establish *BAP1* loss as a reliable predictive biomarker for EZH2 inhibition. Dysregulation of noncoding RNAs also contributes to cell-cycle control, apoptosis, and epithelial–mesenchymal plasticity. For example, promoter hypermethylation–mediated downregulation of the miR-34 family has been reported in mesothelioma, although the clinical utility of these alterations remains unvalidated (50, 53).

Taken together, current evidence indicates that tumor-intrinsic heterogeneity in MPM is jointly determined by combinations of tumor suppressor alterations, clonal architecture, chromosomal instability, and epigenetic state and does not fully overlap with conventional histological subtypes. Alterations involving *BAP1*, *CDKN2A/MTAP*, and the *NF2*/Hippo pathway point to distinct vulnerabilities related to chromatin regulation, cell-cycle–metabolic dependency, and Hippo pathway activation, respectively. However, individual genomic alterations are insufficient to determine transcriptional states, immune ecosystems, or therapeutic responses. Genomic and epigenomic alterations therefore provide a foundation for stratification rather than a complete clinical prediction model, and their functional consequences require further resolution through transcriptomic, translatomic, single-cell, and spatial analyses.

### 2.3 Transcriptional and translational reprogramming shapes malignant cell states

Genomic alterations provide the molecular foundation for tumor evolution in MPM, but binary mutation status alone cannot adequately explain interpatient differences in phenotype and prognosis. Their biological consequences must be translated into observable functional states through transcriptional and translational programs. Malignant cells in MPM are therefore better understood as occupying a continuum of molecular states rather than as fixed entities determined by individual driver events.

Bulk transcriptomic deconvolution studies have shown that the transcriptional profiles of MPM can be decomposed into varying proportions of epithelioid-like and sarcomatoid-like molecular components, forming continuous E-score and S-score distributions, with *CLDN15* and *VIM* representing the respective ends of this continuum. A higher S-score was associated with poorer survival and retained prognostic value within histologically epithelioid MPM (59). Similarly, the morphology-related latent factor LF2 identified by MESOMICS exhibited a continuous distribution (48). Collectively, these findings indicate that histological classification captures the predominant morphological phenotype but does not fully encompass the continuum of transcriptional states in MPM.

Integrated genomic–transcriptomic analyses further showed that the mutation status of *BAP1*, *NF2*, and *SETD2* provided limited prognostic information, whereas transcriptional signatures reflecting downstream functional perturbations showed stronger associations with survival. For example, a high *NF2*-related transcriptional score was associated with poorer survival in the TCGA validation cohort (60). These findings suggest that identical genomic alterations may produce heterogeneous functional consequences. However, the reported associations are prognostic and do not establish that these signatures predict benefit from targeted therapy or immunotherapy.

Translational profiling has also revealed preferential translation of mRNAs involved in ribosome and mitochondrial biogenesis in MPM, accompanied by altered protein synthesis, mitochondrial function, and metabolic output. Combined mTORC1/2 inhibition partially reversed these phenotypes in experimental models (61). The long noncoding RNA *LINC00941* may additionally promote c-MYC synthesis through its interaction with the translation initiation factor eIF4G (62). Nevertheless, the corresponding therapeutic vulnerabilities remain supported primarily by preclinical evidence. Because bulk transcriptomic data represent averaged signals from mixed cell populations, continuous expression gradients may arise from differences in individual-cell states, shifts in subpopulation proportions, or contamination by nonmalignant cells. Single-cell analyses are therefore required to resolve malignant cell subpopulations and their state relationships.

### 2.4 Single-cell resolution of malignant cell heterogeneity

Single-cell RNA sequencing (scRNA-seq) can help determine whether transcriptional gradients observed in bulk tumor samples arise from intrinsic differences in malignant cell states, shifts in the composition of malignant cell populations, or admixture with non-malignant cells. An independent scRNA-seq atlas of human pleural mesothelioma identified multiple tumor cell-intrinsic expression programs, four of which were associated with adverse clinical outcomes. In particular, a sarcomatoid-associated malignant cell program covaried with specific endothelial, myeloid, and T-cell states, and these associations were further supported by imaging analyses and transcriptomic deconvolution in independent cohorts (63). These findings provide independent evidence linking malignant cell states to the broader configuration of the tumor microenvironment. However, the cross-sectional nature of the data precludes determination of the directionality of these relationships.

Complementary evidence was provided by another study integrating patient biopsies with patient-derived organoids. A subset of cells exhibiting fibroblast-like transcriptional features shared genomic rearrangements or copy-number alterations with the corresponding tumor cells, and this relationship was further supported by immunohistochemical validation, suggesting that at least some fibroblast-like cells may originate from malignant cells (64). This finding supports the possibility that mesenchymal-like malignant cell states may be present even in histologically epithelioid tumors. Nevertheless, the small cohort size precluded reliable estimation of their prevalence and clinical significance.

A subsequent single-cell multi-omics study analyzed 93 multisite samples from 40 patients and identified three malignant cell states—epithelioid, sarcomatoid, and “uncommitted”—among malignant cells identified using inferred copy-number profiles (65). Uncommitted cells showed weak epithelioid and sarcomatoid transcriptional features, were enriched for epigenetic regulatory genes including *MEST*, *DNMT3A*, and *TET1*, and were more frequently observed in biphasic tumors. In an independent bulk-transcriptomic cohort of epithelioid MPM, a higher uncommitted-state signature score was associated with poorer survival, although its prognostic value requires prospective validation. Integration of optical genome mapping with scRNA-seq-inferred copy-number profiles further showed that all three malignant cell states could coexist within the same genetic clone (65). These findings suggest that clonal architecture may constrain the emergence of malignant cell states without fully determining their functional phenotype. However, neither the uncommitted state nor the coexistence of multiple cell states within individual clones has yet been independently reproduced in a human single-cell multi-omics cohort using a comparable study design. Moreover, cross-sectional sampling cannot establish whether these states undergo genuine dynamic interconversion.

Cell–cell communication analysis in the same study further implicated TGF-β and GAS6–AXL signaling as candidate regulatory axes of the uncommitted and sarcomatoid states (65). An independent spatial analysis of biphasic MPM showed that sarcomatoid regions were enriched for TGF-β-related, macrophage-associated, and extracellular matrix programs, providing convergent evidence for an association between TGF-β signaling and mesenchymal-like tumor states (66). However, these findings do not independently validate GAS6–AXL signaling and are insufficient to demonstrate that either pathway directly drives malignant-cell state transitions.

Overall, evidence from independent single-cell, organoid, and spatial studies supports a continuum of malignant cell-state heterogeneity in MPM and its association with the surrounding microenvironment. By contrast, the extent to which the uncommitted state is independent of clonal architecture and the identity and functional relevance of its candidate regulatory pathways remain unresolved. Larger prospective cohorts with more balanced histological representation, together with matched multiregional and longitudinal sampling, lineage-tracing approaches, and cell type-specific perturbation experiments, will be required to establish the causal mechanisms underlying malignant cell plasticity and its regulation by the tumor microenvironment.

### 2.5 An integrated model of MPM molecular evolution

The evidence summarized above indicates that MPM does not universally follow a linear evolutionary trajectory characterized by the sequential accumulation of driver events. The principal value of multi-omics integration lies not in compiling additional lists of alterations, but in distinguishing relatively stable events that underlie clonal evolution from variable cell states that reflect functional adaptation, and in determining how these processes interact with microenvironmental selection to shape clinical phenotypes.

Multiregion whole-exome sequencing has provided phylogenetic evidence for the inferred temporal ordering of MPM evolution. An analysis of 90 tumor regions from 22 patients identified phylogenetic structures ranging from relatively linear to highly branched evolution. In this cohort, alterations involving *BAP1*/3p21 and chromosome 4 were classified as early clonal events, homozygous 9p21 deletion occurred exclusively as a clonal event, and *NF2*/22q alterations were predominantly late clonal events, with evidence of parallel evolution (38). Distinct evolutionary clusters were also associated with survival, T-cell infiltration, neoantigen burden, and HLA loss of heterozygosity, suggesting a relationship between clonal architecture and immune selection. However, the study included only 22 surgically treated patients and was enriched for epithelioid MPM. Moreover, evolutionary timing was inferred from multiregion samples collected at a single time point and therefore cannot be assumed to represent all evolutionary trajectories in MPM.

On this basis, we propose a provisional model of molecular evolution in MPM. Environmental exposure and genetic susceptibility promote early clonal genomic alterations, establishing a background of chromatin dysregulation, cell-cycle disruption, and copy-number abnormalities. Subsequent clonal expansion, branched evolution, and the acquisition of selected late events increase genomic heterogeneity. Within this context, epigenetic, transcriptional, and translational adaptations generate a continuum of malignant cell states whose regional distribution is shaped by local immune and stromal selection pressures.

This model links genetic alterations, clonal evolution, cell states, and ecological selection. However, it represents a synthesis of predominantly cross-sectional evidence rather than a validated sequence of fixed temporal stages. Longitudinal sampling, integration of single-cell genomics with spatial multi-omics, and functional perturbation studies will be required to establish the temporal and causal relationships among these biological layers.

## 3 Single-cell and spatial multi-omics dissection of the MPM tumor microenvironment

The genomic, epigenetic, and transcriptional states described in Section 2 shape the intrinsic evolutionary potential of MPM cells but cannot independently explain interpatient differences in immune evasion and therapeutic response. The tumor microenvironment of MPM comprises immune cells, cancer-associated fibroblasts (CAFs), endothelial cells, and the extracellular matrix (ECM), and its biological effects depend on cellular composition and functional states, intercellular interactions, and spatial organization. Single-cell omics can resolve cellular subpopulations and their functional states, whereas spatial multi-omics can further define cellular localization, neighborhood architecture, and local communication. This section therefore examines how the TME contributes to MPM progression and therapeutic response through three interrelated dimensions: immune and stromal cell heterogeneity, spatial niches, and intercellular communication.

### 3.1 Immune-cell composition and functional states

Evidence from immunohistochemistry, transcriptomic deconvolution, and single-cell studies indicates that the immune compartment of MPM is generally characterized by relative myeloid enrichment and substantial interpatient heterogeneity. The extent and functional state of T-cell infiltration vary considerably, whereas B-cell and natural killer (NK)-cell subsets remain less systematically characterized (32, 67–69). Conventional immunohistochemical analyses have identified highly infiltrated tumors containing abundant CD163^+^ macrophages and FOXP3^+^ regulatory T cells, together with increased PD-1 and TIM-3 expression, demonstrating that immune-cell abundance does not necessarily indicate effective antitumor immunity (60, 68). However, restricted marker panels cannot adequately resolve cellular origins or continuous functional states.

Single-cell studies have further revealed myeloid heterogeneity by distinguishing monocyte-derived small peritoneal/pleural macrophages (SPMs) from tissue-resident large peritoneal/pleural macrophages (LPMs). In murine models, SPMs expanded rapidly after tumor implantation and constituted most CD206^+^ M2-like tumor-associated macrophages (TAMs). *Trem2* deficiency reduced this population and delayed tumor progression. By contrast, LPMs exhibited stronger interferon-γ (IFN-γ) responses and contributed to antitumor immune memory, whereas nonselective macrophage depletion impaired immune protection (67). These findings support the need to distinguish myeloid-cell origins and functional states when developing macrophage-directed therapies. Nevertheless, the functional evidence is derived predominantly from preclinical models, and the corresponding populations and therapeutic implications in human MPM remain to be established.

Studies of adaptive immunity suggest that the T-cell receptor (TCR) repertoire provides functional information beyond simple cell counts. In a Chinese mesothelioma cohort, greater baseline TCR diversity and evenness, together with lower clonality, were associated with longer survival; TCR evenness was also associated with outcomes after immune checkpoint inhibitor(ICI) therapy (70). However, the cohort included pleural, peritoneal, and pericardial mesotheliomas, and only a limited number of patients underwent TCR sequencing and had evaluable treatment responses. These findings therefore remain exploratory.

Tumor genomic alterations may also influence immune-cell recruitment. In a study of 89 diffuse pleural mesotheliomas, *CDKN2A* deletion was associated with downregulation of chemokine- and antigen-presentation–related genes and reduced infiltration by CD8^+^ T cells, B cells, and NK cells. Spatial analysis further suggested that immune exclusion was more pronounced in sarcomatoid regions (71). Because these results were obtained from a retrospective association study, they do not establish that *CDKN2A* deletion directly causes an immune-desert phenotype or predicts benefit from immunotherapy.

Collectively, the immune state of MPM is shaped by lymphocyte function, myeloid-cell origin, and the molecular background of the tumor. Current studies are limited by small sample sizes, heterogeneous specimen sources and analytical platforms, and inconsistent definitions of cellular subsets. The proportion of any single immune-cell population should therefore not be regarded as a robust prognostic or predictive biomarker without validation incorporating molecular states, intercellular communication, and spatial neighborhoods.

### 3.2 CAF heterogeneity and extracellular matrix remodeling

In addition to immune cells, CAFs and the ECM are major components of the MPM microenvironment. Single-cell profiling of normal pleura has identified multiple fibroblast subsets (72), but their lineage and functional relationships with CAF states in MPM remain unclear. A single-cell multi-omics study of MPM identified 12 fibroblast subsets. The ratio of *TGFBI*^+^ to *IGF1*^+^ fibroblasts was higher in tumors enriched for uncommitted and sarcomatoid malignant cell states and showed a graded association with poorer survival in an independent cohort of epithelioid pleural mesothelioma (65). These findings link CAF composition to malignant cell state and clinical outcome but do not establish the direction of the relationship. Patient-derived mesothelioma-associated fibroblasts promoted tumor-cell proliferation and migration in co-culture models, supporting a tumor-promoting role for CAFs, although the responsible fibroblast subsets were not identified (73, 74).

ECM features are likewise associated with tumor phenotype and immune infiltration. An integrated analysis of histopathology, multiplex immunofluorescence, and TCGA transcriptomic data showed that high mesothelin expression was accompanied by increased type I collagen, CD8^+^ T cells, and CD68^+^ macrophages. An exploratory TCGA analysis further associated low *MSLN* expression combined with high *COL1A1/COL5A1* expression with poorer survival (75). These findings connect tumor-cell phenotype, immune infiltration, and ECM state but do not demonstrate that collagen deposition directly causes immune exclusion or establish its cellular source. Stromal states may therefore contribute to immunotherapy resistance in MPM, but their spatial organization, functional mechanisms, and predictive value require validation through spatial profiling, functional experiments, and treatment-annotated cohorts.

### 3.3 Spatial organization of tumor, immune, and stromal compartments

Bulk transcriptomics and scRNA-seq can resolve cellular composition and functional states but do not preserve the original spatial relationships among cells. Consequently, they cannot determine whether immune cells penetrate tumor regions or whether distinct cell types form local neighborhoods. Spatial multi-omics remaps cellular identities and molecular states onto tissue architecture, enabling the identification of regional ecological configurations characterized by immune infiltration, immune exclusion, or myeloid–stromal enrichment.

A multiplex immunofluorescence study of 88 MPM and 25 peritoneal mesothelioma samples identified six cellular neighborhoods. Compared with peritoneal mesothelioma, MPM showed more frequent tumor–immune-cell contacts, and tumors with low BAP1 expression exhibited increased contacts between tumor cells and CD8^+^ T cells (76). Multiregion genomic and TCR repertoire analyses further showed that distinct tumor regions from the same patient could share major driver alterations, whereas most T-cell clones remained restricted to individual regions (77). In human tumor tissues, shorter distances between malignant cells and marker-defined M2a- or M2c-like macrophages were also associated with poorer survival (78). Xenograft models additionally demonstrated distinct regional distributions of neutrophil-like and monocyte/macrophage-like cells (79). Together, these findings indicate that neither overall cellular abundance nor a shared genomic background can substitute for local spatial assessment. However, predefined marker panels, restricted tissue sampling, and reliance on animal models limit causal interpretation and generalizability.

Spatial TME organization also varies in parallel with malignant cell states. GeoMx digital spatial profiling of 139 morphologically defined regions from eight biphasic MPMs revealed continuous transcriptional differences among epithelioid, transitional, and sarcomatoid regions. Sarcomatoid regions exhibited enhanced ECM remodeling, angiogenesis, and immune-cell infiltration, together with enrichment of HAVCR2–LGALS9 signaling and *TGFB1*/M2-like macrophage signatures (66). Xenium *in situ* analysis further confirmed that epithelioid, uncommitted, and sarcomatoid malignant cells could occur adjacent to or intermingled with one another within the same tumor, and that morphologically epithelioid regions could contain uncommitted malignant cells (65).

GeoMx measures mixed expression signals within morphologically selected regions and therefore cannot definitively identify the cellular sources of individual transcripts. Conversely, the Xenium analysis used a predefined targeted gene panel, making it more suitable for *in situ* validation of previously identified cell states than for the unbiased discovery of unknown spatial niches. Both studies were also small and cross-sectional. Their findings therefore support spatial covariation between malignant cell states and TME remodeling but do not establish temporal ordering or causality.

High-dimensional spatial proteomic analyses provide complementary evidence. Imaging mass cytometry combined with WES identified multiple tumor-cell populations even in cohorts composed predominantly of histologically epithelioid MPM. Cell clusters with sarcomatoid-like features were associated with distinct immune-cell compositions and poorer survival (80). These results further demonstrate that histological subtype does not fully represent the local tumor–immune ecosystem. However, the limited cohort size necessitates independent validation of the reproducibility and prognostic value of these spatially defined cell clusters.

Spatial niches may also be associated with therapeutic response. A conference abstract describing a neoadjuvant immunotherapy cohort reported that pretreatment tumors from responders more frequently contained immune-activated neighborhoods composed of CD8^+^ effector-memory T cells and B-cell aggregates. By contrast, nonresponders showed enrichment of TAMs, *SFRP2*^+^ CAFs, and TGF-β–associated immunosuppressive stromal structures (81). Because these findings were derived from baseline specimens and reported only in abstract form, they should be regarded as exploratory associations between pre-existing ecological states and treatment response. Overall, spatial studies have revealed the coexistence of local cellular contacts and ecological states. Nevertheless, spatial proximity is only a prerequisite for cellular communication, and the direction and functional consequences of the underlying signals require experimental validation.

### 3.4 Cell–cell communication networks contribute to immune evasion

Functional studies have provided initial evidence for signaling from malignant cells to myeloid populations. Mesothelioma-derived granulocyte–macrophage colony-stimulating factor (GM-CSF) induced granulocytes to generate NOX2-dependent reactive oxygen species, thereby suppressing T-cell proliferation and activation. Neutralization of GM-CSF or inhibition of reactive oxygen species partially restored T-cell function (82, 83). Certain malignant cell populations may also recruit neutrophils through CXCL5. Neutrophil-derived histamine can subsequently promote tumor-cell migration through histamine receptor H1 and induce *CXCL5*, *CXCL8*, and *CSF3* expression, generating a positive-feedback loop that sustains myeloid-cell recruitment (84). These findings demonstrate that the secretory state of malignant cells can influence the composition and function of local myeloid populations, although the direct contribution of this circuit to adaptive immune suppression remains unclear.

Tumor-intrinsic states may alter the secretome, whereas recruited myeloid cells may reciprocally influence malignant phenotypes. In epithelioid MPM, low *DKK3* expression was associated with promoter hypermethylation. In experimental models, restoration of *DKK3* expression reduced the release of tumor-derived extracellular vesicles and CSF1, decreased the formation of immunosuppressive TAMs, and increased CD8^+^ T-cell infiltration (85). Conversely, TAM-derived GPNMB may interact with CD44 on tumor cells to promote proliferation and the maintenance of stem-like properties. Host *Gpnmb* deficiency and CD44 blockade both reduced tumor burden in experimental models (86). These findings support a bidirectional circuit involving tumor secretory reprogramming, myeloid recruitment, and feedback reinforcement of malignant phenotypes, although the relative contribution of each component in human MPM has not been quantified.

Single-cell and spatial analyses have additionally proposed TGF-β and GAS6–AXL as candidate communication axes (65, 66). However, these inferences are based primarily on co-expression, ligand–receptor analysis, and spatial proximity and therefore do not establish signaling direction or causal state transitions. Immune evasion in MPM is consequently better conceptualized as a multicellular feedback network than as the additive effect of several independent pathways. Establishing the direction, functional consequences, and clinical actionability of these communication circuits will require cell type-specific perturbation, spatial validation of ligand–receptor activity, and longitudinal analysis of paired pre- and post-treatment samples.

### 3.5 From local cellular states to an integrated tumor ecosystem

Collectively, current evidence indicates that TME heterogeneity in MPM encompasses at least four interrelated but noninterchangeable dimensions: cellular composition, functional state, spatial organization, and intercellular communication (65, 67, 68, 76). These dimensions should be interpreted jointly rather than in isolation. The most appropriate analytical unit is therefore not an individual cell type or signaling pathway, but the local ecological configuration formed by malignant cells, immune cells, and stromal components within a defined tissue region.

These ecological configurations exhibit pronounced regional heterogeneity. Distinct lesions from the same patient—or different regions within a single tumor—may share major driver alterations while displaying divergent T-cell clonal compositions, malignant cell states, and immune–stromal architectures (65, 66, 77). Patient-level mean expression profiles or single-site biopsies may therefore obscure local differences with potential therapeutic relevance. Immune activation, myeloid dominance, and immune exclusion are accordingly better regarded as potentially coexisting ecological states than as fixed, mutually exclusive clinical subtypes.

Accordingly, Section 4 organizes recurrent immune configurations reported across MPM cohorts into a provisional framework, while treating tumor-intrinsic molecular states and stromal–spatial features as explanatory modifiers.

## 4 A proposed multi-omics–informed immune-ecosystem framework for MPM stratification

### 4.1 Rationale for moving beyond histology

Epithelioid, sarcomatoid, and biphasic histological subtypes remain fundamental to the pathological diagnosis and prognostic assessment of MPM. However, histology primarily captures the predominant morphological features within the sampled region and cannot simultaneously represent the tumor’s molecular state, immune ecosystem, and spatial heterogeneity. Moving beyond histology therefore does not imply abandoning its clinical value, but rather incorporating complementary dimensions of disease stratification.

Multi-omics studies provide direct evidence for this complementarity. Epithelioid–sarcomatoid transcriptional signatures form a continuum and provide additional prognostic information within histologically epithelioid MPM (59). Single-cell analyses further demonstrate that epithelioid, uncommitted, and sarcomatoid malignant cell states can coexist within the same tumor (65). MESOMICS showed that WHO histological classification explained only approximately 2%–10% of interpatient variation across different molecular layers, with a mean of approximately 6%. Other dimensions identified in that study, including ploidy, adaptive immunity, and the CpG island methylator phenotype (CIMP), were not equivalent to the morphology-related axis (48). Furthermore, alterations in *BAP1*, *NF2*, and *CDKN2A/MTAP* occur across histological subtypes (32–34), and their binary status does not adequately represent downstream functional states or microenvironmental phenotypes (48, 60).

Spatial heterogeneity further limits the representativeness of single-site biopsies. Different regions from the same patient may exhibit discordant *NF2* alterations, regionally restricted T-cell clones, and divergent immune states (37, 66, 76, 77). A local specimen should therefore be regarded as a spatial snapshot rather than a complete representation of the patient-level tumor ecosystem.

**Figure 1:**
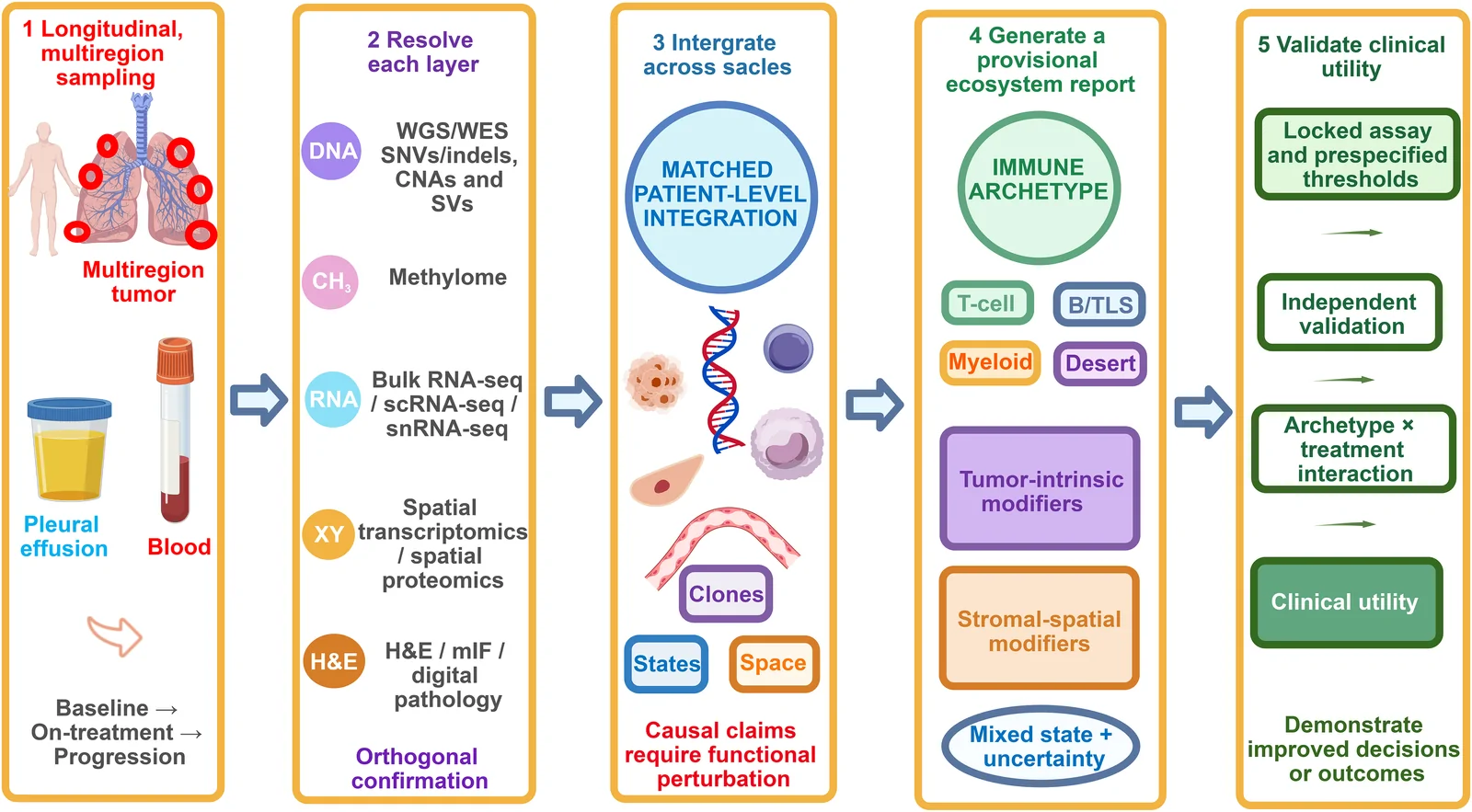
Proposed multi-omics integration workflow for MPM stratification and clinical translation. Longitudinal and multiregion tumor, pleural-effusion, and blood specimens are characterized using genomic, epigenomic, bulk and single-cell or single-nucleus transcriptomic, spatial, and histopathologic approaches. Matched patient-level integration links clonal architecture, malignant and nonmalignant cell states, and spatial niches, whereas causal claims require functional perturbation. The resulting provisional ecosystem report combines a candidate immune archetype with tumor-intrinsic and stromal–spatial modifiers while explicitly recording mixed states and uncertainty. Clinical translation requires a locked assay with prespecified thresholds, independent external validation, archetype-by-treatment interaction testing, and evidence that the tool improves clinical decisions or patient outcomes. This workflow is conceptual and does not represent a clinically validated classifier. MPM, malignant pleural mesothelioma; WGS, whole-genome sequencing; WES, whole-exome sequencing; SNV, single-nucleotide variant; indel, insertion/deletion; CNA, copy-number alteration; SV, structural variant; scRNA-seq, single-cell RNA sequencing; snRNA-seq, single-nucleus RNA sequencing; mIF, multiplex immunofluorescence; H&E, hematoxylin and eosin. The schematic was created by the authors using BioGDP.com (87) and the Generic Diagramming Platform (GDP).

On this basis, we propose a staged, multi-omics–informed workflow for MPM stratification and clinical translation, spanning longitudinal and multiregion sampling, matched cross-scale integration, provisional ecosystem reporting, and clinical validation [**Figure 1**, created with BioGDP.com (87)]. Within this workflow, recurrent patterns of immune-cell composition, functional state, and spatial organization reported across published human MPM cohorts are organized into four provisional archetypes: T-cell–inflamed, B-cell/tertiary lymphoid structure (TLS)–organized, myeloid-dominant, and immune-desert. The evidentiary basis, candidate markers, and operational limitations of these archetypes are detailed below.

### 4.2 Provisional immune-ecosystem archetypes

Because no existing study has jointly clustered genomic, single-cell, and spatial data within a unified MPM cohort, the four archetypes proposed here were not derived by reclustering original patient-level data. Instead, they represent a conceptual synthesis of recurrent immune patterns reported across published human MPM cohorts. Patil et al. identified T-cell–enriched, B-cell/antigen-presentation–enriched, and immune-desert phenotypes in 87 MPM samples (88). Rigutto et al. subsequently distinguished T-cell–enriched, B-cell–enriched, and myeloid-enriched groups using high-dimensional single-cell profiling and spatial imaging (69). MESOMICS further indicated that adaptive and innate immune states vary along continuous axes rather than forming sharply separated categories (48).

**Figure 2:**
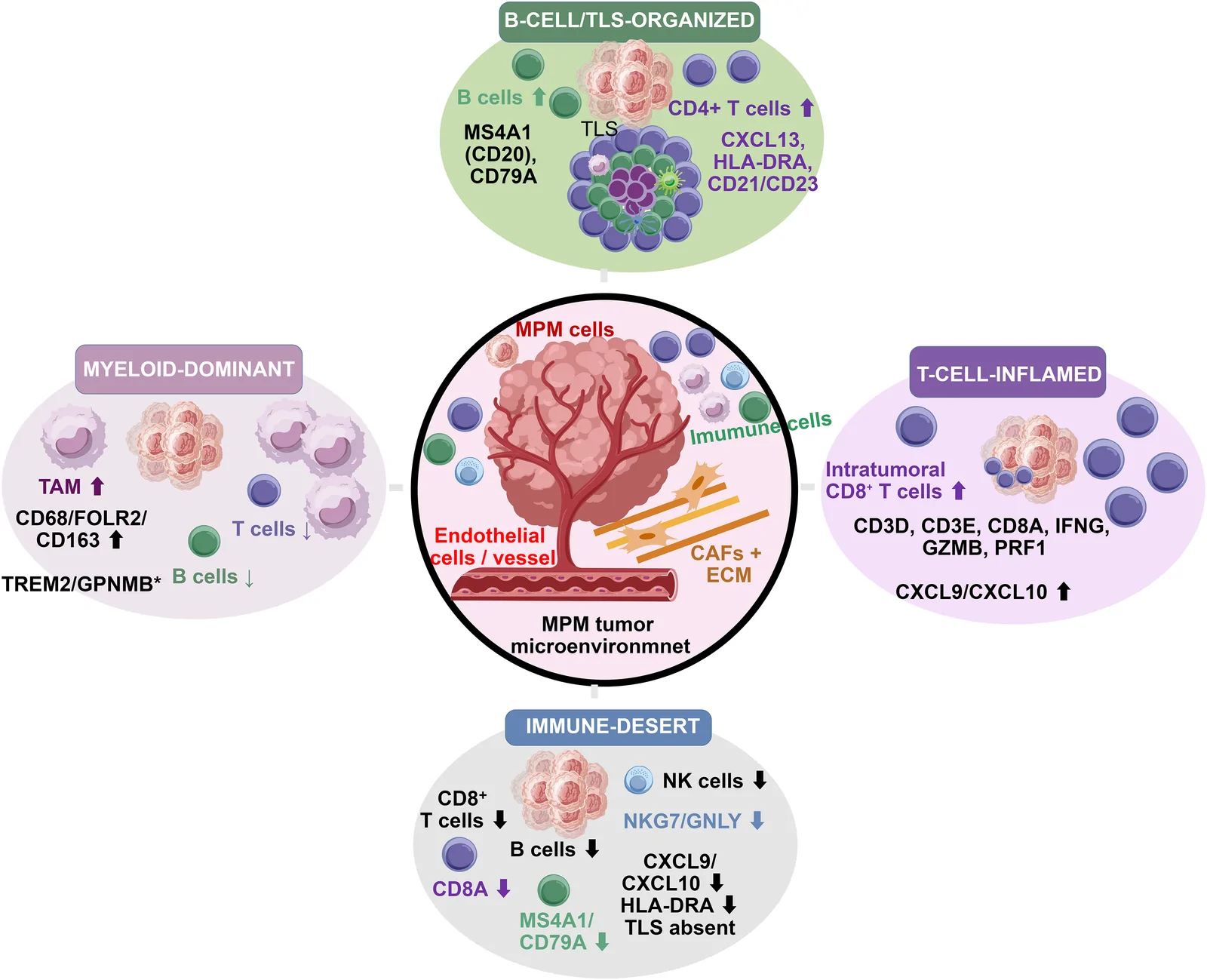
Four provisional immune-ecosystem archetypes in MPM. The T-cell–inflamed archetype is characterized by intratumoral CD8^+^ T-cell enrichment together with interferon-related chemokine and cytotoxic-effector programs. The B-cell/TLS-organized archetype is characterized by B-cell and CD4^+^ T-cell enrichment, antigen-presentation signals, and spatially organized TLS. The myeloid-dominant archetype is characterized by TAM enrichment accompanied by relative T- and B-cell depletion, whereas the immune-desert archetype shows reduced T-, B-, and NK-cell infiltration, attenuated chemokine and antigen-presentation signals, and a lack of detectable TLS. The central schematic illustrates the major components of the MPM tumor microenvironment, including malignant cells, immune cells, endothelial cells, CAFs, and ECM. Cell numbers are schematic and indicate relative enrichment or depletion within individual study cohorts rather than absolute cell density. Cell populations not shown in a given archetype are not necessarily absent but do not form part of its proposed operational definition. TREM2 and GPNMB are exploratory TAM-state markers rather than mandatory defining criteria. Tumor-intrinsic molecular states and stromal–spatial features are treated as modifiers rather than additional categories. These literature-derived archetypes are nonexclusive, are not clinically validated subtypes, and currently lack standardized cross-platform thresholds. MPM, malignant pleural mesothelioma; TLS, tertiary lymphoid structure; TAM, tumor-associated macrophage; NK, natural killer; CAF, cancer-associated fibroblast; ECM, extracellular matrix. The schematic was created by the authors using BioGDP.com (87) and the Generic Diagramming Platform (GDP).

Accordingly, the four archetypes should be regarded as provisional and potentially overlapping ecological configurations rather than fixed, mutually exclusive clinical subtypes. Each archetype should be operationalized using a composite module incorporating cellular composition, functional markers, and spatial features rather than any individual biomarker. No standardized cross-platform scores or classification thresholds are currently available, and individual tumors may exhibit mixed or intermediate states. The cellular configurations, candidate markers, and evidence boundaries of the four proposed immune-ecosystem archetypes are summarized schematically in **Figure 2** [Created with BioGDP.com (87)] and operationally in **Table 1**.

**Table 1:** Operational definitions, supporting evidence, and translational interpretation of the four provisional immune-ecosystem archetypes in MPM.

Proposed archetype	Candidate biomarkers and spatial definition	Supporting evidence	Evidence boundaries
T-cell–inflamed	Increased *CD3D/CD3E*, *CD8A*, *IFNG*, *CXCL9/CXCL10*, and *GZMB/PRF1*; CD8^+^ T-cell infiltration into the tumor parenchyma. *PDCD1*, *LAG3*, and *TIGIT* describe exhaustion but do not independently define the archetype.	T-cell–enriched group (88); adaptive-immunity–high state in MESOMICS (48); functional T-cell analysis in PROMISE-meso (90).	Candidate enrichment state for prospective ICI biomarker studies. Total CD8^+^ T-cell abundance and broad inflammatory phenotypes lack stable predictive value. BAP1 is an associated modifier rather than a defining marker (89).
B-cell/TLS–organized	Increased *MS4A1* (CD20), *CD79A*, *CD74*, *HLA-DRA*, and *CXCL13*; spatial aggregation of CD20^+^ B cells and CD4^+^ T cells; CD21 and CD23 may help assess TLS maturation.	B-cell/antigen-presentation–enriched group (88); MATCH study (91); single-cell identification and spatial validation of TLSs (69).	Supported primarily as a prognostic state. Its ability to predict greater benefit from ICIs than from alternative treatment has not been established.
Myeloid-dominant	Increased CD68, FOLR2, and CD163, with a reduced T/B-cell-to-myeloid-cell ratio. TREM2, STAB1, LAIR1, GPNMB, and MARCO are exploratory state markers.	Myeloid-enriched group (69); CCL2-mediated recruitment (92); TAM origin and function (67); spatial and functional evidence (78, 86).	Support studies of selective TAM reprogramming or myeloid-directed therapy combined with ICIs. TAM heterogeneity, including the treatment interaction reported for PD-1^+^CD68^+^ macrophages (90), argues against nonselective macrophage depletion.
Immune-desert	Low *CD3D/CD3E*, *CD8A*, *MS4A1/CD79A*, *NKG7/GNLY*, *CXCL9/CXCL10*, and *HLA-DRA*; absence of effective lymphocyte infiltration and TLSs within the tumor and its periphery, without clear myeloid predominance.	Immune-desert group identified by Patil et al. (88); immune depletion associated with *CDKN2A* deletion (71); spatial classification in PROMISE-meso (90).	Provide a rationale for studying immune priming or restoration of chemotactic signaling before ICI therapy. *CDKN2A* is not a defining marker, and distinction from myeloid dominance or immune exclusion remains sensitive to sampling and marker coverage.

“Increased” and “low” indicate relative differences within individual study cohorts; no absolute cross-platform thresholds have been established. These archetypes are literature-derived, potentially overlapping, and not validated treatment-selection categories. CAF/ECM features, immune exclusion, and tumor-intrinsic molecular states should be recorded as modifiers rather than additional classes. CAF, cancer-associated fibroblast; ECM, extracellular matrix; ICI, immune checkpoint inhibitor; MPM, malignant pleural mesothelioma; TAM, tumor-associated macrophage; TLS, tertiary lymphoid structure.

#### 4.2.1 T-cell–inflamed archetype

The T-cell–inflamed archetype is characterized by coordinated increases in T-cell recruitment, effector activity, and inflammatory signaling, together with histological confirmation that CD8^+^ T cells have entered the tumor parenchyma. Its principal classification basis is provided by the T-cell–enriched group identified by Patil et al. and the adaptive-immunity–high state reported by MESOMICS (48, 88). Inflammatory features associated with low BAP1 expression may serve as a molecular modifier but are not required to define this archetype (89).

In the PROMISE-meso cohort, PD-1^+^CD8^+^ T cells were associated with longer progression-free survival among pembrolizumab-treated patients, whereas total CD8^+^ T-cell abundance and broadly defined inflamed phenotypes did not demonstrate stable predictive value (90). The T-cell–inflamed archetype may therefore serve as a candidate enrichment state in prospective ICI biomarker studies but should not currently be used as an established criterion for treatment selection.

#### 4.2.2 B-cell/TLS–organized archetype

This archetype is characterized by both enhanced B-cell/antigen-presentation signals and their spatial organization, including the formation of B-cell–T-cell aggregates or TLSs. Patil et al. initially identified a B-cell/antigen-presentation–enriched immune group (88). Subsequent independent evidence from the MATCH study and Rigutto et al. associated CD20^+^ B cells, higher *MS4A1/CXCL13* expression, and TLS enrichment with longer survival (69, 91).

The B-cell/TLS–organized archetype therefore has relatively consistent prognostic support and a biological context compatible with ICI activity. However, existing evidence does not demonstrate that TLSs predict greater benefit from ICIs than from alternative treatments. Their treatment-predictive value requires validation in randomized therapeutic cohorts.

#### 4.2.3 Myeloid-dominant archetype

The myeloid-dominant archetype is characterized by the predominance of myeloid populations accompanied by relative depletion of T and B cells. Rigutto et al. directly identified a myeloid-dominant group enriched in total TAMs and FOLR2^+^ TAMs, with reduced T- and B-cell abundance and poorer outcomes (69). Studies of pleural effusions, single-cell datasets, and spatially resolved tissues further support CCL2-mediated monocyte recruitment, functional divergence among macrophages of different origins, and associations between specific TAM states and tumor progression (67, 78, 86, 92).

Importantly, not all myeloid populations are immunosuppressive. In PROMISE-meso, PD-1^+^CD68^+^ macrophages were associated with benefit from PD-1 blockade, and the association was stronger when these cells co-occurred with PD-1^+^CD8^+^ T cells (90). We therefore use “myeloid-dominant” rather than “myeloid-immunosuppressive.” Potential therapeutic strategies include selective reprogramming or targeting of tumor-promoting TAM states, such as TREM2^+^ or GPNMB^+^ populations, in combination with ICIs. Nonselective depletion of all macrophages is not supported by the available evidence, and selective myeloid-directed strategies remain predominantly preclinical.

#### 4.2.4 Immune-desert archetype

The immune-desert archetype is characterized by broadly reduced T-, B-, and NK-cell infiltration, attenuated chemotactic and antigen-presentation signals, and an absence of TLSs. Its principal basis is the immune-desert group identified by Patil et al. (88). *CDKN2A* deletion has been associated with downregulation of inflammatory chemokines and antigen-presentation genes, reduced lymphocyte infiltration, and more pronounced immune depletion in sarcomatoid regions (71). However, these findings remain associative, and *CDKN2A* status should not be considered a defining requirement for this archetype.

The immune-desert archetype must also be distinguished from myeloid dominance and immune exclusion. Immune-desert tumors lack effective immune infiltration; myeloid-dominant tumors retain abundant TAMs despite relative lymphocyte depletion; and immune-excluded tumors contain immune cells that are predominantly retained at the tumor margin or within stromal regions. Although PROMISE-meso classified tumors into desert, excluded, and inflamed spatial phenotypes, these broad categories did not predict benefit from pembrolizumab (90). The limited immunological basis for ICI monotherapy in immune-desert tumors suggests that immune priming, restoration of chemotactic signaling, or epigenetic modulation combined with ICIs could be explored, but these strategies remain unvalidated hypotheses.

These four archetypes are classified according to their predominant immune configuration. *BAP1*, *CDKN2A/MTAP*, *NF2*, CIMP, malignant cell states, CAF/ECM features, and immune-exclusion patterns may explain heterogeneity within each archetype but do not constitute additional categories. Future studies should first establish whether the four immune archetypes can be reproducibly identified across platforms and cohorts and then determine whether molecular and spatial features provide independent or interactive information.

### 4.3 Validation strategies and clinical implications

Validation of the proposed framework should address three sequential questions: whether the archetypes can be reproducibly identified across cohorts and analytical platforms, whether they provide information independent of histology and existing biomarkers, and whether they predict differential benefit from specific treatments.

First, tissue sampling, pathological assessment, analytical platforms, and clinical outcome definitions should be standardized in prospective multicenter MPM cohorts. Prespecified targeted RNA and multiplex immunofluorescence panels could be used to quantify T-cell, B-cell/TLS, myeloid, and immune-desert modules. Representative samples should undergo single-cell and spatial profiling to validate cellular origins and spatial localization. Multiregion sampling subsets should additionally determine the sensitivity of archetype assignment to biopsy location (65, 76, 77).

Second, scoring algorithms and classification thresholds should be developed and locked within a training cohort before evaluation in independent external cohorts. Validation should assess intercenter reproducibility, discrimination, and calibration, as well as incremental value beyond disease stage, histological subtype, PD-L1 expression, and individual genomic alterations. Model development, validation, and reporting should follow the REMARK and TRIPOD recommendations (93, 94). Tumor-intrinsic molecular states and stromal–spatial features should be prespecified as covariates or potential interaction terms rather than used *post hoc* to generate additional categories.

Finally, prognostic associations must be distinguished from treatment-predictive effects. Prespecified prospective–retrospective analyses of archived specimens from completed randomized trials could provide an initial validation step. Subsequent prospective treatment cohorts or biomarker-stratified trials should formally test archetype-by-treatment interactions (95). Real-world data may provide complementary association evidence but cannot independently establish treatment-selection utility. The proposed framework should progress from a hypothesis-generating model to a clinical stratification tool only after demonstrating cross-platform reproducibility, independent treatment-predictive value, and measurable improvement in clinical decision-making.

## 5 Multi-omics-guided precision therapeutics

Systemic treatment for MPM has expanded from chemotherapy alone to dual immune checkpoint blockade and immunochemotherapy. CheckMate 743 established the clinical value of nivolumab plus ipilimumab in unresectable MPM (11), while the phase III IND.227 trial subsequently demonstrated improved overall survival with the addition of pembrolizumab to platinum–pemetrexed chemotherapy (96). Nevertheless, no multi-omics classifier has been validated for routine treatment selection in MPM. The immediate value of multi-omics lies primarily in identifying candidate vulnerabilities, explaining therapeutic heterogeneity, and informing biomarker-enriched clinical trials. To avoid equating biological plausibility with clinical efficacy, the following therapeutic strategies are discussed according to three evidence levels: established clinical evidence, early human exploratory evidence, and preclinical or hypothesis-generating evidence.

### 5.1 Targeting tumor-intrinsic vulnerabilities

MPM lacks frequent activating driver alterations with established matched therapies. Its therapeutic vulnerabilities arise more commonly from synthetic-lethal or epigenetic dependencies created by tumor-suppressor loss. The association between BAP1 inactivation and dysregulated PRC2/EZH2 activity provides a biological rationale for EZH2 inhibition. In a single-arm phase II study of relapsed or refractory MPM enriched for immunohistochemically defined BAP1 inactivation, tazemetostat achieved a 12-week disease control rate of 54%, although objective responses were infrequent (58). Because nearly all enrolled tumors were BAP1-inactivated and no BAP1-retained comparator group was available, the study supports preliminary clinical activity of EZH2 inhibition but does not establish BAP1 loss as a treatment-predictive biomarker.

Co-deletion of *CDKN2A* and *MTAP* creates a metabolic–epigenetic dependency. *MTAP* loss leads to methylthioadenosine accumulation, creating selective vulnerabilities to MTA-cooperative PRMT5 inhibitors and MAT2A inhibition (40, 42, 43). Early basket trials have demonstrated clinical activity of MTA-cooperative PRMT5 inhibitors in *MTAP*-deleted solid tumors (97), while MAT2A inhibition has also entered phase I clinical evaluation (98). However, MPM-specific efficacy, the optimal method for determining *MTAP* status, and mechanisms of resistance remain undefined. *MTAP* loss is therefore more appropriately used as an enrichment criterion for clinical trials than as an established treatment-selection biomarker in MPM.

*NF2* loss/Hippo signaling provides another potential therapeutic direction involving YAP/TAZ–TEAD inhibition. This approach has progressed into clinical evaluation, with the TEAD palmitoylation inhibitor VT3989 showing preliminary antitumor activity in a phase I/II trial of refractory mesothelioma (99). However, multiregion analyses have shown that *NF2* alterations may be subclonal and spatially heterogeneous (37). Moreover, responses to VT3989 were not confined to tumors with detectable *NF2* alterations, indicating that genomic inactivation alone may not adequately reflect pathway dependence. Trials of Hippo pathway–directed therapies should therefore integrate *NF2*/Merlin status with YAP/TEAD transcriptional activity, clonal abundance, and interlesional concordance.

Interventions targeting miR-503 (100), UHRF1 (52), mTOR-associated translational regulation (101), or epigenetic silencing of *WIF1* (50) remain supported predominantly by cell-based or animal studies. These mechanisms warrant continued investigation as candidate vulnerabilities but should not be presented at the same evidentiary level as strategies that have entered clinical evaluation.

### 5.2 Optimizing immunotherapy and cell-based therapy according to immune-ecosystem state

The four immune-ecosystem archetypes proposed in Section 4 provide testable hypotheses for treatment matching but cannot currently guide clinical treatment selection. T-cell–inflamed and B-cell/TLS–organized tumors may retain a relatively intact basis for immune priming. Myeloid-dominant tumors may require selective reprogramming of tumor-promoting myeloid states, whereas immune-desert tumors may first require restoration of antigen presentation, chemotactic signaling, and immune-cell recruitment. These proposed relationships have not been tested through archetype-by-treatment interaction analyses in randomized trials and should therefore not be applied to current clinical decision-making.

As summarized in Sections 4.2.1 and 4.2.3, the PROMISE-meso findings suggest that functionally defined immune-cell states may be more informative than total immune-cell abundance or broad spatial classifications (90). These observations support biomarker-enriched prospective studies but do not establish current treatment-selection criteria. Similarly, the co-expression of *LAG3* with *CD8A* and *PDCD1* may reflect adaptive inhibitory feedback following immune infiltration rather than independently demonstrating the efficacy of LAG-3 blockade (102). Strategies targeting the NKG2A–HLA-E and TIGIT axes likewise remain at the mechanistic or early translational stage in MPM (63, 103). Evidence supporting stimulator of interferon genes (STING)-directed strategies is more indirect. Activation of the cyclic GMP–AMP synthase (cGAS)–STING pathway has enhanced immune checkpoint therapy in non-MPM preclinical tumor models (104), while tumor-cell STING activation has also been reported to prime NK-cell therapy (105). These findings provide a rationale for investigating STING-based combinations in MPM but should not be interpreted as direct evidence of therapeutic efficacy in this disease or as evidence supporting chimeric antigen receptor–engineered natural killer-cell (CAR-NK) therapy.

Multi-omics profiling may also help optimize target-antigen selection and characterize microenvironmental barriers to cellular therapy. Preclinical work has explored universal umbilical cord blood-derived mesothelin-targeted chimeric antigen receptor T (CAR-T) cells, providing early evidence of the feasibility and antitumor potential of this platform (106). Separately, a phase I study of intrapleurally administered mesothelin-targeted CAR-T cells followed by pembrolizumab demonstrated feasibility and preliminary antitumor activity in malignant pleural disease, predominantly MPM (107). However, the small, nonrandomized design precluded determination of the incremental contribution of pembrolizumab relative to CAR-T-cell therapy alone. Future studies should evaluate not only mesothelin expression but also its intratumoral spatial heterogeneity, treatment-emergent antigen loss, T-cell trafficking, and myeloid–stromal suppression.

### 5.3 Targeting microenvironment-mediated immune exclusion and resistance

Myeloid cells and CAF/ECM states may cooperatively maintain immune exclusion in MPM, although individual myeloid and fibroblast populations do not necessarily perform equivalent functions. Single-cell studies suggest that therapeutic strategies should selectively reprogram or target tumor-promoting TAM states rather than broadly depleting all macrophages (67). TREM2, GPNMB–CD44, and CCL2-associated signaling represent candidate intervention nodes (67, 86, 92), but there is currently no therapeutic evidence demonstrating that targeting these pathways improves clinical outcomes in human MPM. The ability of oncolytic measles virus to induce type I interferon–associated responses in macrophages further demonstrates the potential therapeutic plasticity of myeloid states (108). CAF activation, collagen deposition, and TGF-β signaling may jointly generate an immune-excluding stroma in a subset of MPMs. *SFRP2*^+^ CAFs, the *TGFBI*^+^/*IGF1*^+^ fibroblast ratio, and ECM-associated markers could serve as exploratory enrichment variables (65, 75, 81). However, current evidence is derived primarily from observational single-cell and spatial analyses and does not establish that CAF- or TGF-β–directed therapy improves ICI benefit in MPM. Such combinations should preferentially be evaluated in patients with stromal enrichment and clearly defined spatial immune exclusion rather than applied without biomarker selection.

Therapeutic perturbation studies provide proof of concept for TME plasticity. Drug screening in patient-derived cells identified enhanced tumor control with entinostat plus anti–PD-1 therapy in immunocompetent models, although the *in vitro* screening system lacked intact immune and stromal compartments (109). In animal models, hyperthermic intrathoracic chemotherapy (HITOC) increased CD8^+^ T-cell infiltration, reduced M2-like TAMs, and upregulated immune checkpoint expression, suggesting a potential rationale for combination with PD-1 or CTLA-4 blockade (110, 111). These findings remain preclinical and do not demonstrate equivalent ecological remodeling or clinical benefit in patients with MPM.

Overall, multi-omics has not yet brought MPM into an era in which omics results directly determine treatment. Its near-term translational role is to incorporate *BAP1*/EZH2, *MTAP*/PRMT5, *NF2*/Hippo, provisional immune archetypes, and stromal–myeloid features as prespecified enrichment variables in prospective trials. Paired pre- and post-treatment samples should then be used to assess target modulation, ecosystem transitions, and the evolution of therapeutic resistance. The candidate selection markers, therapeutic strategies, and current evidence boundaries are summarized in **Table 2**.

**Table 2:** Candidate multi-omics–informed therapeutic strategies and their current evidence boundaries in MPM.

Candidate molecular or ecological state	Candidate selection variables	Potential therapeutic strategy and evidence	Translational interpretation and evidence boundary
BAP1–PRC2/EZH2 dysregulation	BAP1 protein loss or genomic inactivation; exploratory CIMP- or PRC2-associated states (48, 58).	EZH2 inhibition; evaluated in a single-arm phase II study in MPM (58)	Disease control supports biological tractability, but the absence of a BAP1-retained comparator prevents validation of BAP1 loss as a treatment-predictive biomarker.
*CDKN2A/MTAP* co-deletion	Homozygous *MTAP* deletion or loss of MTAP protein expression (33, 40, 43)	MTA-cooperative PRMT5 or MAT2A inhibition; phase I evidence in molecularly selected advanced malignancies (42, 97, 98).	May support trial enrichment; MPM-specific efficacy, optimal MTAP testing, and resistance mechanisms remain undefined.
*NF2*/Hippo dysregulation	*NF2* inactivation, YAP/TAZ–TEAD transcriptional activity, and clonal abundance (37, 44, 45)	TEAD palmitoylation inhibition, exemplified by VT3989, first-in-human, single-arm phase I/II clinical evidence (99).	Preliminary activity justifies further study, but *NF2* alteration alone is not a validated predictive biomarker and may be affected by spatial heterogeneity.
T-cell–inflamed or B-cell/TLS–organized archetype	Intratumoral CD8^+^ T cells, IFN-γ signaling, or TLSs; detailed markers are provided in **Table 1** (69, 88, 90, 91)	ICIs and immunotherapy combinations; Phase III evidence supports ICI efficacy (11, 96)	These immune states may be used as prospective stratification hypotheses, but their treatment-predictive value has not been validated.
Myeloid-dominant archetype	CD68, FOLR2, and CD163 expression; TREM2^+^, GPNMB^+^, or other TAM states (67, 69, 86, 92)	Selective TAM reprogramming or myeloid-directed therapy combined with ICIs; observational and preclinical evidence (67, 69, 86, 90)	TAMs are functionally heterogeneous; current evidence does not support nonselective macrophage depletion.
Immune-desert archetype	Low lymphocyte, chemotactic, and antigen-presentation modules (71, 88, 90)	Immune priming or restoration of chemotactic signaling followed by ICI therapy; hypothesis-generating and preclinical evidence	The biological basis for ICI monotherapy may be limited, but this archetype cannot currently guide treatment selection (71, 88, 90).
Mesothelin expression with relative antigen preservation	Mesothelin expression, spatial heterogeneity, and post-treatment antigen retention	Mesothelin-targeted CAR-T cells combined with ICIs; Phase I study (107)	Remains experimental; antigen loss and TME-mediated suppression must be considered.
CAF/ECM enrichment or immune exclusion	*SFRP2*^+^ CAFs, *TGFBI*^+^/*IGF1*^+^ fibroblast ratio, collagen deposition, and TGF-β–associated features (65, 66, 75, 81)	CAF-, TGF-β–, or ECM-modulating therapy combined with ICIs; observational and preclinical evidence.	These combinations should be evaluated in biomarker-selected, stroma-enriched populations rather than applied indiscriminately.

Except for systemic treatments established in randomized trials, all treatment–biomarker relationships presented in this table represent candidate stratification strategies or clinical-trial hypotheses and do not constitute current recommendations for routine MPM treatment.

## 6 Challenges and future perspectives

### 6.1 Technical and data-integration challenges

As a rare malignancy, MPM presents substantial difficulties in obtaining high-quality fresh tissue, multiregion samples, and paired pre- and post-treatment specimens. Although BEAT-MESO has generated a large-scale single-nucleus RNA-sequencing atlas from 179 formalin-fixed, paraffin-embedded samples with adequate tumor content (112), single-cell and spatial studies incorporating multiregion sampling, spatial validation, or comprehensive treatment outcomes generally include only a few dozen patients or fewer (65). Limited sample sizes and uneven representation of histological subtypes restrict the detection of rare cellular states and treatment interactions while increasing the risks of overfitting and inadequate external validation.

Different analytical platforms are subject to distinct and sometimes opposing technical biases. Single-cell RNA sequencing (scRNA-seq) is affected by expression dropout, dissociation-induced stress, selective cell loss, and batch effects (113, 114). Single-nucleus RNA sequencing (snRNA-seq) is applicable to frozen and selected fixed tissues but may capture cytoplasmic transcripts and certain functional states less efficiently. Spatial technologies require a trade-off between transcriptomic coverage and spatial resolution: capture spot–based platforms may combine signals from multiple cells, whereas imaging-based platforms generally rely on predefined targeted panels (65, 112, 115). Cross-platform integration therefore cannot rely solely on computational label transfer and requires orthogonal validation using shared markers, spatial proteomics, and histopathological assessment.

Functional models likewise cannot fully reconstruct the TME. Patient-derived cell screening can provide high-throughput drug-sensitivity data, but prolonged culture may select subclones adapted to *in vitro* conditions and eliminate immune, stromal, and spatial interactions (109). Organoid–immune co-culture systems and immunocompetent animal models may recover selected functional components but cannot substitute for evidence from patients receiving treatment.

A more fundamental challenge is that cross-omics associations do not by themselves establish temporal or causal relationships. Future studies should integrate genomic, single-cell, and spatial data from matched multiregion and longitudinal specimens. Cell type-specific perturbation, lineage tracing, and paired pre- and post-treatment analyses will be required to distinguish the relative contributions of clonal evolution, cellular state changes, and microenvironmental selection.

### 6.2 Barriers to clinical translation

Comprehensive single-cell and spatial analyses require high-quality tissue, multiple analytical platforms, and specialized bioinformatics pipelines. Their cost, turnaround time, and sample requirements remain incompatible with routine clinical practice. A more feasible translational pathway may involve compressing complex omics signatures into targeted RNA panels, multiplex immunofluorescence assays, immunohistochemical panels, or digital pathology scores. However, these simplified tools must demonstrate concordance with the original omics-defined states in independent cohorts. RNA-based signals cannot be assumed to represent corresponding protein expression or spatial architecture.

Clinical implementation will also require standardized pre-analytical procedures, quality-control criteria, model versions, and reporting formats, together with solutions for intercenter reproducibility, data governance, and interpretability. For a rare tumor such as MPM, robust model development and validation are unlikely to be achieved within a single institution. Multicenter collaborative networks and shared reference specimens will therefore be needed to support external quality assessment.

As discussed in Section 4.3, any ecosystem-based score must demonstrate incremental value beyond disease stage, histological subtype, and existing biomarkers before clinical adoption. Treatment-annotated cohorts must also distinguish prognostic associations from treatment-predictive effects. Even a complex model with high internal accuracy would not justify routine implementation unless it altered treatment decisions or improved patient outcomes.

### 6.3 Toward dynamic precision oncology in MPM

Precision treatment of MPM may ultimately evolve from static assessment based on a single biopsy toward longitudinal, multimodal monitoring. This approach, however, remains investigational.

Digital pathology and artificial intelligence may integrate tissue morphology with genomic and immune–spatial features. Existing MPM studies indicate that whole-slide images can capture stromal, inflammatory, and cellular-diversity features associated with survival (116). Nevertheless, digital pathology has not been shown to reproducibly identify the immune archetypes proposed in this review or to substitute for spatial omics. In the near term, such models are more appropriately positioned as lower-cost screening or research-enrichment tools and will require external multicenter validation, calibration, and interpretability analyses.

Liquid biopsy may complement repeated tissue sampling, although different analytes capture distinct biological information. Circulating tumor DNA (ctDNA) primarily reflects tumor burden and selected clonal genomic alterations, and preliminary studies have demonstrated the feasibility of detecting tumor-specific variants in MPM (117). Extracellular-vesicle RNA and circulating proteins may provide indirect functional information, but their cellular origins and analytical stability remain insufficiently defined. Pleural effusions may enrich for local tumor and immune components but cannot reconstruct their original spatial relationships within tissue. Liquid biopsy is therefore better suited to longitudinal monitoring and the identification of emerging resistance than to replacing tissue-based ecosystem classification.

Because ecosystem states may evolve during treatment, prospective trials should stratify patients according to baseline ecosystem state and use paired tumor tissue, pleural effusion, and blood samples to evaluate treatment-associated changes and their relationships with response and resistance. “Ecosystem-state–adapted” treatment can progress from a conceptual model to an adaptive therapeutic strategy only after dynamic monitoring has been shown to improve treatment modification and patient outcomes.

## 7 Conclusions

Multi-omics studies have generated three interconnected insights into the heterogeneity of MPM. First, genomic events involving *BAP1*, *CDKN2A/MTAP*, and *NF2*/Hippo impose evolutionary constraints and create candidate vulnerabilities but do not independently determine functional phenotypes or therapeutic responses (32, 33, 37, 48). Second, epithelioid, uncommitted, and sarcomatoid malignant cell states can coexist within the same genetic clone (65), supporting a contribution from nongenetic regulation to state formation. Third, immune and stromal features exhibit substantial spatial heterogeneity (65, 66, 76), indicating that local ecosystem architecture provides information not captured by bulk profiles or single-site sampling. Histological classification therefore remains the foundation of diagnosis, whereas multi-omics provides complementary functional and ecological information.

Based on recurrent immune patterns reported across human cohorts, we propose four provisional immune-ecosystem archetypes: T-cell–inflamed, B-cell/TLS–organized, myeloid-dominant, and immune-desert. Tumor-intrinsic molecular states and stromal–spatial features are treated as explanatory modifiers within these archetypes (48, 69, 88). This literature-derived framework remains hypothesis-generating rather than clinically validated and is intended to guide prospective biomarker and treatment-interaction studies.

At present, the principal clinical value of multi-omics in MPM lies in mechanistic discovery, the generation of stratification hypotheses, and the design of biomarker-enriched trials rather than the direct selection of therapy. Multicenter standardized testing, independent external validation, randomized treatment-interaction analyses, and longitudinal sampling will be required to determine whether molecular and ecological features have reproducible treatment-predictive value. MPM classification can progress from a predominantly histology-based system toward molecular–immune ecosystem–informed precision therapy only when complex omics information can be translated into robust, interpretable, scalable tools that demonstrably improve clinical decision-making.
